# CT-based radiomics nomograms for preoperative prediction of diffuse-type and signet ring cell gastric cancer: a multicenter development and validation cohort

**DOI:** 10.1186/s12967-022-03232-x

**Published:** 2022-01-24

**Authors:** Tao Chen, Jing Wu, Chunhui Cui, Qinglie He, Xunjun Li, Weiqi Liang, Xiaoyue Liu, Tianbao Liu, Xuanhui Zhou, Xifan Zhang, Xiaotian Lei, Wei Xiong, Jiang Yu, Guoxin Li

**Affiliations:** 1 Department of General Surgery & Guangdong Provincial Key Laboratory of Precision Medicine for Gastrointestinal Tumor, Nanfang Hospital, The First School of Clinical Medicine, Southern Medical University, Guangzhou, 510515 Guangdong China; 2 Department of General Surgery, Zhujiang Hospital, Southern Medical University, Guangzhou, 510280 Guangdong China; 3 Department of The First Clinical Medical College, Southern Medical University, Guangzhou, 510515 China; 4 School of Biomedical Engineering, Southern Medical University, Guangzhou, 510515 Guangdong Province China; 5 Medical Imaging Center, Nanfang Hospital, Southern Medical University, No.1838, North Guangzhou Avenue, Guangzhou, 510515 Guangdong China

**Keywords:** Gastric cancer, Pathology, Radiomics, Support vector machine, Nomogram

## Abstract

**Background:**

The prevalence of diffuse-type gastric cancer (GC), especially signet ring cell carcinoma (SRCC), has shown an upward trend in the past decades. This study aimed to develop computed tomography (CT) based radiomics nomograms to distinguish diffuse-type and SRCC GC preoperatively.

**Methods:**

A total of 693 GC patients from two centers were retrospectively analyzed and divided into training, internal validation and external validation cohorts. Radiomics features were extracted from CT images, and the Lauren radiomics model was established with a support vector machine (SVM) classifier to identify diffuse-type GC. The Lauren radiomics nomogram integrating radiomics features score (Rad-score) and clinicopathological characteristics were developed and evaluated regarding prediction ability. Further, the SRCC radiomics nomogram designed to identify SRCC from diffuse-type GC was developed and evaluated following the same procedures.

**Results:**

Multivariate analysis revealed that Rad-scores was significantly associated with diffuse-type GC and SRCC (*p* < 0.001). The Lauren radiomics nomogram showed promising prediction performance with an area under the curve (AUC) of 0.895 (95%CI, 0.957–0.932), 0.841 (95%CI, 0.781–0.901) and 0.893 (95%CI, 0.831–0.955) in each cohort. The SRCC radiomics nomogram also showed good discrimination, with AUC of 0.905 (95%CI,0.866–0.944), 0.845 (95%CI, 0.775–0.915) and 0.918 (95%CI, 0.842–0.994) in each cohort. The radiomics nomograms showed great model fitness and clinical usefulness by calibration curve and decision curve analysis.

**Conclusion:**

Our CT-based radiomics nomograms had the ability to identify the diffuse-type and SRCC GC, providing a non-invasive, efficient and preoperative diagnosis method. They may help guide preoperative clinical decision-making and benefit GC patients in the future.

**Supplementary Information:**

The online version contains supplementary material available at 10.1186/s12967-022-03232-x.

## Introduction

Gastric cancer (GC) is the fifth most common cancer and the third leading cause of cancer-related death worldwide [1]. Although the overall incidence of GC has significantly decreased over recent decades, the incidence of Lauren diffuse-type GC is constantly rising, and the predominant increase occurred in the signet ring cell carcinoma (SRCC) [2]. From 1973 to 2000, the incidence of SRCC increased from 0.1 to 1.4 cases per 100,000 persons as recorded in the Surveillance, Epidemiology, and End Results (SEER) database [3].

The Lauren classification and the World Health Organization (WHO) classification systems are the mainstream histological classification methods for GC [4]. The Lauren classification divides GC into intestinal-type, diffuse-type and mixed-type according to the histological morphology and cell characteristics of GC [5]. According to the WHO classification system, GC with at least 50% signet-ring cells (SRC) in the pathological specimen is defined as SRCC [6]. Although all SRCCs are classified as Lauren diffuse type [7–9], they have distinct etiology, pathogenesis, prognosis and tumor biological behavior, such as lymph node metastasis rate, chemosensitivity [10–15]. If diffuse-type and SRCC GC can be diagnosed and distinguished in an early stage, it will be of great help to the choice of treatment schemes and the prognosis evaluation.

In clinical practice, endoscopic biopsies are generally used to provide doctors with a reliable pathological diagnosis of GC. However, studies revealed that the Lauren classification’s consistent rate between biopsy and surgical samples was only 64.7% [16], and there was often a high false-negative rate when dealing with diffuse infiltrating-type GC [17]. Computed tomography (CT) is the most commonly used imaging modality for diagnosing and assessing the staging of gastric malignancies. However, traditional CT based on lesion distribution, wall thickness and enhancement pattern has limitations in diagnosing the diffuse type of gastric carcinoma [18]. Meanwhile, 18F-FDG PET/CT has low sensitivity in detecting SRCC [19].

Recently, radiomics, as a typical case of medical application of machine learning that extracts quantitative features from radiological images and builds a signature for the complete characterization of tumors, has exhibited great potential in improving diagnostic, prognostic, and predictive accuracy [20, 21]. Research has shown that radiomics could be a useful tool for identifying occult peritoneal metastasis in patients with advanced GC [22] and differentiating Borrmann type IV GC from primary gastric lymphoma [23]. However, there are limited studies to explore the possibility of radiomics in identifying diffuse-type and SRCC gastric cancer [24].

Thus, we conducted this study to develop a CT-based radiomics nomogram, providing a noninvasive and efficient preoperative diagnosis method to identify diffuse-type and SRCC GC. In the future, it may help guide preoperative clinical decision-making and benefit GC patients.

## Materials and methods

### Patients

This retrospective study was approved by the institutional review board of two medical centers, and the need for informed patient consent was waived. All procedures performed involving human participants were following the 1964 Helsinki Declaration and its later amendments. Patients who underwent total or partial radical gastrectomy and histologically confirmed GC between December 2007 and March 2016 were enrolled. The detailed inclusion criteria were as follows: (1) patients who underwent surgery for GC; (2) patients who underwent standard contrast-enhanced CT less than 15 days before surgery; (3) patients with complete clinicopathologic data. Patients who received neoadjuvant chemotherapy (NAC) therapy or radiotherapy before surgery were excluded to avoid the influence of these factors on the tumor size and degree of invasion. The demographic and clinicopathologic data of patients, including age, sex, tumor site, tumor size (maximum diameter), CEA, CA199, Lauren classification, Borrmann classification, differentiation and tumor stage, were obtained from medical records. Tumor staging was performed based on the American Joint Committee on Cancer tumor-node-metastasis (TNM) Staging Manual, 8th Edition.

Flow diagrams for eligible patients were shown in Additional file 1: Figure S1. Finally, a total of 693 patients (453 males and 240 females; mean age, 56.38 ± 11.85 years; age range, 22–87 years) from 2 medical centers were enrolled in the study, including 587 patients from center 1 (Nanfang Hospital of Southern Medical University, Guangzhou, China) and 106 patients from center 2 (Zhujiang Hospital, Guangzhou, China). To develop a Lauren radiomics model to identify diffuse-type GC, we divided all patients into three cohorts: one training cohort (n = 300 from center 1), one internal validation cohort (n = 287 from center 1) and one external validation cohort (n = 106 from center 2) (Additional file 1: Figure S1a). Moreover, the SRCC radiomics model was designed to identify SRCC from diffuse-type GC. A total of 443 diffuse-type GC patients were included and divided into three cohorts: one training cohort (n = 280 from center 1), one internal validation cohort (n = 114 from center 1) and one external validation cohort (n = 49 from center 2) (Additional file 1: Figure S1b). The sample size consideration was shown in Additional file 1: S1.

### CT image acquisition and radiomics feature extraction

The procedures of CT image acquisition and retrieval were described in detail in Additional file 1: S2. Then CT images were exported to the ITK-SNAP 3.6 (ITK-SNAP 3.X TEAM) software, and three-dimensional (3D) segmentation of the region of interest (ROI) was performed (Additional file 1: Figure S2). The algorithms for tumor ROIs delineation and reproducibility evaluation of intraobserver (reader 1 twice) and interobserver (reader 1 vs. reader 2) were described in Additional file 1: S3. The preprocessing was applied to the ROI images with different parameters (Additional file 1: Table S1) to enrich features before extracting the texture features (Additional file 1: S4). Then we applied the feature extraction method to the ROI in MATLAB 2016b (Mathworks), and series of texture features were generated from the images (Additional file 1: Table S2**)**. Then the feature values were preprocessed with a filtering feature selection method (Additional file 1: S5**)**.

### Feature selection, construction and evaluation of the radiomics SVM models

We used the Relief forward selection (RFS) algorithm [25] and an exhaustive test based on the performance of the SVM classifier (Additional file 1: S6) to find feature subset with the best distinguishing characteristics for the radiomics model [25]. The SVM model was based on the LIBSVM software package developed by Professor Lin et al. in 2001 (https://www.csie.ntu.edu.tw/~cjlin). A high penalty parameter c could effectively improve the model’s accuracy, but an excessive high penalty parameter would cause over-fitting status. The range of c was limited to prevent this situation, and the tenfold cross-validation and grid search method was applied to find the best combination of SVM model parameters (c and g) (Additional file 1: Figure S3). Then all extracted features were ranked from the most important to the least important, and different feature sets were obtained using the exhaustive test from the ordered sequence 1 ≤ m ≤ M. The set of first m features was fed into the SVM classifier. Its performance for differentiating different GC types was evaluated by receiver operating characteristic (ROC) curves and the area under the curve (AUC). The detailed steps of feature selection were shown in Additional file 1: S7 in the Supplement. Differences in the AUC values between the three cohorts were assessed using the Delong test. The pathological classification radiomics feature score obtained in SVM models of each patient was seen as Rad-score. Kaplan–Meier survival analyses were used to estimate the difference in 5-year disease-free survival (DFS) and 5-year overall survival (OS) between the high Rad-score and low Rad-score groups.

### Development and evaluation of radiomics nomograms

Multivariate logistic regression was applied to select independent predictors of diffuse-type and SRCC GC from the clinical characteristics. The significant predictors among the clinical characteristics and the Rad-score were entered into the logistic regression analysis to develop the radiomics nomogram. The diagnostic performance and calibration of the radiomics nomogram were evaluated based on ROC and calibration curves. Decision curve analysis was applied to assess the clinical usefulness of the radiomics nomograms by quantifying the net benefit at different threshold probabilities.

### Statistical analysis

Analyses were performed using SPSS version 26.0 (SPSS Inc., Chicago, IL, USA). Continuous variables were presented as the mean ± standard deviation and compared with the t-test. Categorical variables were expressed as frequency (percentage) and compared with Chi-squared tests or Fisher's exact test as appropriate. Nomograms and calibration curves were generated with the rms package of R software (version 4.0.3; R Foundation for Statistical Computing, Vienna, Austria). A *p*-value of < 0.05 was set as the threshold for statistical significance.

## Results

### Clinical characteristics of all patients

A total of 693 patients (453 males and 240 females; mean age, 56.38 ± 11.85 years; age range, 22–87 years) were included in the study. The clinicopathologic characteristics of the assessed patients were listed in Table 1. Clinical characteristics, including tumor location, differentiation status, Borrmann type, levels of CEA and CA199, and TNM stages were significantly different between intestinal-type and diffuse-type GC patients.

**Table 1:** Clinicopathologic characteristics of all patients enrolled

Clinical characteristics	Lauren radiomics model								
	Training cohort (*n* = 300)			Validation cohort (*n* = 287)			External validation cohort (*n* = 106)		
	Intestinal type (*n* = 150)	Diffuse type (*n* = 150)	*p* value	Intestinal type (*n* = 43)	Diffuse type (*n* = 244)	*p* value	Intestinal type (*n* = 57)	Diffuse type (*n* = 49)	*p* value
Gender, *n*			0.146			0.040			0.355
Male	104 (69.3)	92 (61.3)		35 (81.4)	160 (65.6)		31 (54.4)	31 (63.3)	
Female	46 (30.7)	58 (38.7)		8 (18.6)	84 (34.4)		26 (45.6)	18 (36.7)	
Age, mean ± SD, years	57.91 ± 10.97	54.95 ± 11.54	0.437	58.26 ± 10.56	54.11 ± 11.27	0.414	59.86 ± 12.72	61.71 ± 14.97	0.104
Age, *n*			0.160			0.032			0.478
< 60	81 (54.0)	93 (62.0)		22 (51.2)	166 (68.0)		24 (42.1)	24 (49.0)	
≥ 60	69 (46.0)	57 (38.0)		21 (48.8)	78 (32.0)		33 (57.9)	25 (51.0)	
Tumor size, cm	2.84 ± 1.54	3.27 ± 1.89	0.034	2.57 ± 1.84	3.44 ± 2.05	0.308	—	—	—
Tumor size, *n*			0.076			0.049			—
< 4 cm	113 (75.3)	99 (66.0)		33 (76.7)	149 (66.5)		—	—	
≥ 4 cm	37 (24.7)	51 (34.0)		10 (23.3)	95 (33.5)		—	—	
Tumor location, *n*			0.017			0.003			0.977
Upper	35 (23.3)	16 (10.7)		15 (34.9)	36 (14.8)		16 (28.1)	14 (28.6)	
Middle	22 (14.7)	27 (18.0)		2 (4.7)	41 (16.8)		15 (26.3)	12 (24.5)	
Lower	78 (52.0)	82 (54.7)		24 (48.8)	135 (55.3)		26 (45.6)	23 (46.9)	
Whole	15 (10.0)	25 (16.7)		2 (4.7)	32 (13.1)		0	0	
Differentiation status, *n*			< 0.001			< 0.001			0.009
Well	17 (11.3)	1 (0.7)		6 (14.0)	1 (0.4)		6 (10.5)	0 (0)	
Moderate	61 (40.7)	6 (4.0)		20 (46.5)	11 (4.5)		21 (36.8)	11 (22.4)	
Poor and undifferentiated	72 (48.0)	143 (95.3)		17 (39.5)	232 (95.1)		30 (52.6)	38 (77.6)	
Borrmann type, *n*			< 0.001			0.029			0.177
1	16 (10.7)	8 (5.3)		6 (14.0)	18 (7.4)		16 (28.1)	14 (28.6)	
2	47 (31.3)	19 (12.7)		15 (34.9)	49 (20.1)		29 (50.9)	16 (32.7)	
3	83 (55.3)	110 (73.3)		19 (44.2)	132 (54.1)		11 (19.3)	17 (34.7)	
4	4 (2.7)	13 (8.7)		3 (7.0)	45 (18.4)		1 (1.8)	2 (4.1)	
CEA, *n*			< 0.001			0.007			0.009
Elevated	43 (28.7)	73 (48.7)		11 (25.6)	117 (48.0)		14 (24.6)	24 (49.0)	
Normal	107 (71.3)	77 (51.3)		32 (74.4)	127 (52.0)		43 (75.4)	25 (51.0)	
CA199, *n*			< 0.001			0.008			0.518
Elevated	44 (29.3)	72 (48.0)		11 (25.6)	116 (47.5)		11 (19.3)	12 (24.5)	
Normal	106 (70.7)	78 (52.0)		32 (74.4)	128 (52.5)		46 (80.7)	37 (75.5)	
Depth of invasion, *n*			< 0.001			< 0.001			0.421
T1	40 (26.7)	17 (11.3)		17 (39.5)	32 (13.1)		9 (15.8)	3 (6.1)	
T2	25 (16.7)	16 (10.7)		8 (18.6)	18 (7.4)		79 (12.3)	5 (10.2)	
T3	13 (8.7)	22 (14.7)		3 (7.0)	21 (8.6)		10 (17.5)	9 (18.4)	
T4	72 (48.0)	95 (63.3)		15 (34.9)	173 (70.9)		31 (54.4)	32 (65.3)	
Lymph node metastasis, *n*			< 0.001			< 0.001			0.019
N0	78 (52.0)	43 (28.7)		34 (79.1)	70 (28.7)		22 (38.6)	9 (18.4)	
N1	18 (12.0)	29 (19.3)		3 (7.0)	55 (22.5)		14 (24.6)	7 (14.3)	
N2	23 (15.3)	25 (16.7)		3 (7.0)	45 (18.4)		10 (17.5)	16 (32.7)	
N3	31 (20.7)	53 (35.3)		3 (7.0)	74 (30.3)		11 (19.3)	17 (34.7)	
Distant metastasis, *n*			0.310			0.536			0.237
M0	147 (98.0)	144 (96.0)		41 (95.3)	237 (97.1)		52 (91.2)	41 (83.7)	
M1	3 (2.0)	6 (4.0)		2 (4.7)	7 (2.9)		5 (8.8)	8 (16.3)	
TNM stage, *n*			< 0.001			< 0.001			0.082
I	54 (36.0)	24 (16.0)		22 (51.2)	37 (15.2)		14 (24.6)	4 (8.2)	
II	38 (25.3)	29 (19.3)		12 (27.9)	46 (18.9)		12 (21.1)	8 (16.3)	
III	46 (30.7)	75 (50.0)		7 (16.4)	131 (53.6)		26 (45.6)	29 (59.2)	
IV	12 (8.0)	22 (14.7)		2 (4.7)	30 (12.3)		5 (8.8)	8 (16.3)	

### Feature selection and construction of the Lauren radiomics SVM model

**Fig. 1:**
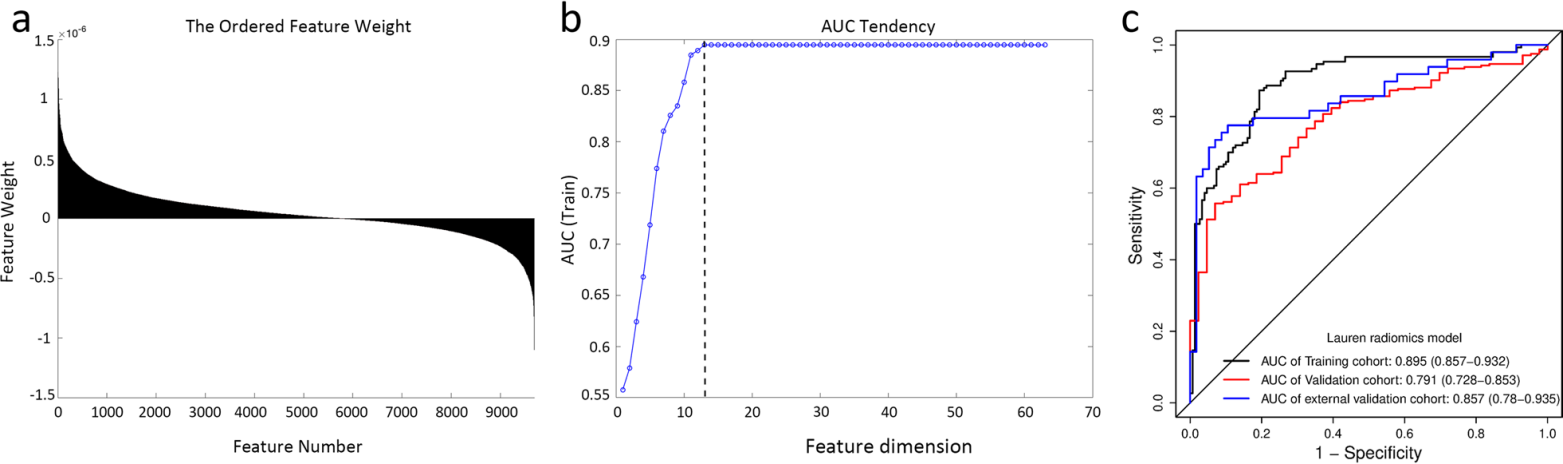
Construction of the Lauren radiomics SVM model. **a** The weight ordering of 9691 radiomics features. **b** The optimal feature subset of the Lauren radiomics SVM model included 13 features. **c** ROC curves of Lauren radiomics SVM model in the training, internal validation and external validation cohorts. *ROC* receiver operator characteristic, *SVM* Support vector machine

A total of 9691 features were extracted from the tumor ROI with satisfactory interobserver and intraobserver reproducibility assessments (Additional file 1: S8). The weight ordering of radiomics features was obtained by the Relief algorithm (Fig. 1a). The feature subset with the best discrimination ability for the radiomics model was obtained using the exhaustive test based on the performance of the support vector machine (SVM) classifier. Finally, the optimal feature subset with 13 features achieved excellent performance in distinguishing Lauren diffuse-type and intestinal-type GC (Fig. 1b and Additional file 1: Table S3), yielding AUC values of 0.895 (95% confidence interval (CI) 0.957–0.932), 0.791 (95%CI0.728–0.853) and 0.857 (95%CI0.78–0.935) in the training, internal validation and external validation cohort, respectively (Fig. 1c). Multivariate analysis revealed that Rad-score was the significant predictor between intestinal-type and diffuse-type GC (OR, 4.164; 95%CI, (3.121,5.557); *p* < 0.001) (Additional file 1: Table S4). Further, statistical difference was found in terms of 5-year DFS and OS between the high Rad-score group (diffuse-type) and low Rad-score group (intestinal-type) (Additional file 1: S9**)**.

### Construction and evaluation of the Lauren radiomics nomograms

**Fig. 2:**
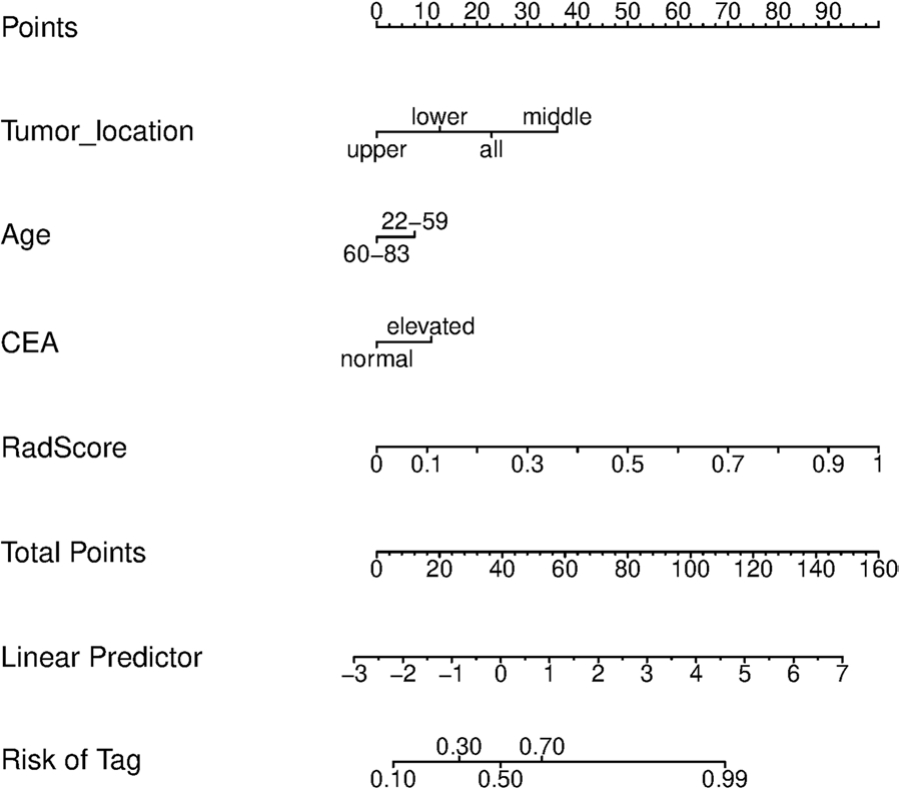
Development of the Lauren radiomics nomograms

Univariate logistic regression analyses showed that age, tumor size, tumor location, and elevated CEA and CA199 had statistically significant *p*-values between the diffuse-type and intestinal-type GC patients. Multivariate logistic analysis revealed that age (OR, 0.979 [0.958, 1.000], *p* = 0.049), tumor location (OR, 1.347 [1.035, 1.753], *p* = 0.027) and elevated CEA (OR, 2.302 [1.417, 3.740], *p* = 0.001) were independent predictors (Table 2). The Rad-score and clinical characteristics were incorporated to build the Lauren radiomics nomogram (Fig. 2).

**Table 2:** Clinicopathologic characteristics of patients with diffuse-type GC

Clinical characteristics	SRCC radiomics model								
	Training cohort (n = 280)			Validation cohort (n = 114)			External validation cohort (n = 49)		
	Non-SRCC (n = 180)	SRCC (n = 100)	p value	Non-SRCC (***n* = 59)**	SRCC (***n* = 55)**	p value	Non-SRCC (n** = 34)**	SRCC (***n* = 15)**	p** value**
Gender, *n*			0.004			0.098			0.109
Male	125 (69.4)	52 (52.0)		43 (72.9)	32 (58.2)		24 (70.6)	7 (46.7)	
Female	55 (30.6)	48 (48.0)		16 (27.1)	23 (41.8)		10 (29.4)	8 (53.3)	
Age, mean ± SD, years	55.32 ± 10.61	53.36 ± 11.93	0.198	56.39 ± 11.70	51.36 ± 11.87	0.914	63.38 ± 15.39	57.93 ± 13.71	0.574
Age, *n*			0.220			0.241			0.004
< 60	111 (61.7)	69 (69.0)		38 (64.4)	41 (74.5)		12 (35.3)	12 (80.0)	
≥ 60	69 (38.3)	31 (31.0)		21 ( (35.6)	14 (25.5)		22 (64.7)	3 (20.0)	
Tumor size, cm	3.29 ± 1.87	3.15 ± 2.29	0.032	3.74 ± 2.24	3.27 ± 2.10	0.839		–	–
Tumor size, *n*			0.433			0.231			–
< 4 cm	114 (63.3)	68 (68.0)		31 (52.5)	35 (63.6)		–	–	
≥ 4 cm	66 (36.7)	32 (32.0)		28 (47.5)	20 (36.4)		–	–	
Tumor location, *n*			0.099			0.046			0.008
Upper	27 (15.0)	7 (7.0)		14 (23.7)	4 (7.3)		13 (38.2)	1 (6.7)	
Middle	34 (18.9)	14 (14.0)		10 (16.9)	10 (18.2)		10 (29.4)	2 (13.3)	
Lower	98 (54.4)	62 (62.0)		29 (49.2)	28 (50.9)		11 (32.4)	12 (80.0)	
Whole	21 (11.7)	17 (17.0)		6 (10.2)	13 (23.6)		0 (0)	0 (0)	
Differentiation status, *n*			0.162			0.049			0.079
Well	2 (1.1)	0 (0)		0 (0)	0 (0)		0 (0)	0 (0)	
Moderate	11 (6.1)	2 (2.0)		4 (6.8)	0 (0)		10 (29.4)	1 (6.7)	
Poor and undifferentiated	167 (92.8)	98 (98.0)		55 (93.2)	55 (100)		24 (70.6)	14 (93.3)	
Bormann type, *n*			0.001			< 0.001			0.506
1	11 (6.1)	7 (7.0)		5 (8.5)	4 (7.3)		10 (29.4)	4 (26.7)	
2	28 (15.6)	15 (15.0)		16 (27.1)	8 (14.5)		13 (38.2)	3 (20.0)	
3	120 (66.7)	48 (48.0)		37 (62.7)	25 (45.5)		10 (29.4)	7 (46.7)	
4	21 (11.7)	30 (30.0)		1 (1.7)	18 (32.7)		1 (2.9)	1 (6.7)	
CEA, *n*			0.775			0.995			0.404
Elevated	86 (47.8)	46 (46.0)		30 (50.8)	28 (50.9)		18 (52.9)	6 (40.0)	
Normal	94 (52.2)	54 (54.0)		29 (49.2)	27 (49.1)		16 (47.1)	9 (60.0)	
CA199, *n*			0.655			0.851			0.094
Elevated	86 (47.8)	45 (45.0)		30 (50.8)	27 (49.1)		6 (17.6)	6 (40.0)	
Normal	94 (52.2)	55 (55.0)		29 (49.2)	28 (50.9)		28 (82.4)	9 (60.0)	
Depth of invasion, *n*			0.295			0.282			0.200
T1	21 (11.7)	19 (19.0)		7 (11.9)	8 (14.5)		1 (2.9)	2 (13.3)	
T2	15 (8.3)	6 (6.0)		5 (8.5)	4 (7.3)		2 (5.9)	3 (20.0)	
T3	23 (12.8)	9 (9.0)		2 (3.4)	7 (12.7)		7 (20.6)	2 (13.3)	
T4	121 (67.2)	66 (66.0)		45 (76.3)	36 (65.5)		24 (70.6)	8 (53.3)	
Lymph node metastasis, *n*			0.043			0.395			0.878
N0	50 (27.8)	32 (32.0)		16 (27.1)	18 (32.7)		7 (20.6)	2 (13.3)	
N1	42 (23.3)	13 (13.0)		15 (25.4)	13 (23.6)		5 (14.7)	2 (13.3)	
N2	38 (21.1)	15 (15.0)		13 (22.0)	6 (10.9)		10 (29.4)	6 (40.0)	
N3	50 (27.8)	40 (40.0)		15 (25.4)	18 (32.7)		12 (35.3)	5 (33.3)	
Distant metastasis, *n*			0.337			0.518			0.707
M0	175 (97.2)	95 (95.0)		58 (98.3)	53 (96.4)		28 (92.4)	13 (86.7)	
M1	5 (2.8)	5 (5.0)		1 (1.7)	2 (3.6)		6 (17.6)	2 (13.3)	
TNM stage, *n*			< 0.001			0.043			0.653
I	25 (13.9)	22 (22.0)		8 (13.6)	10 (18.2)		2 (5.9)	2 (13.3)	
II	37 (20.6)	14 (14.0)		12 (20.3)	9 (16.4)		6 (17.6)	2 (13.3)	
III	103 (57.2)	40 (40.0)		36 (61.0)	26 (47.3)		20 (58.9)	9 (60.0)	
IV	15 (8.3)	24 (24.0)		3 (5.1)	10 (18.2)		6 (17.6)	2 (13.3)

**Fig. 3:**
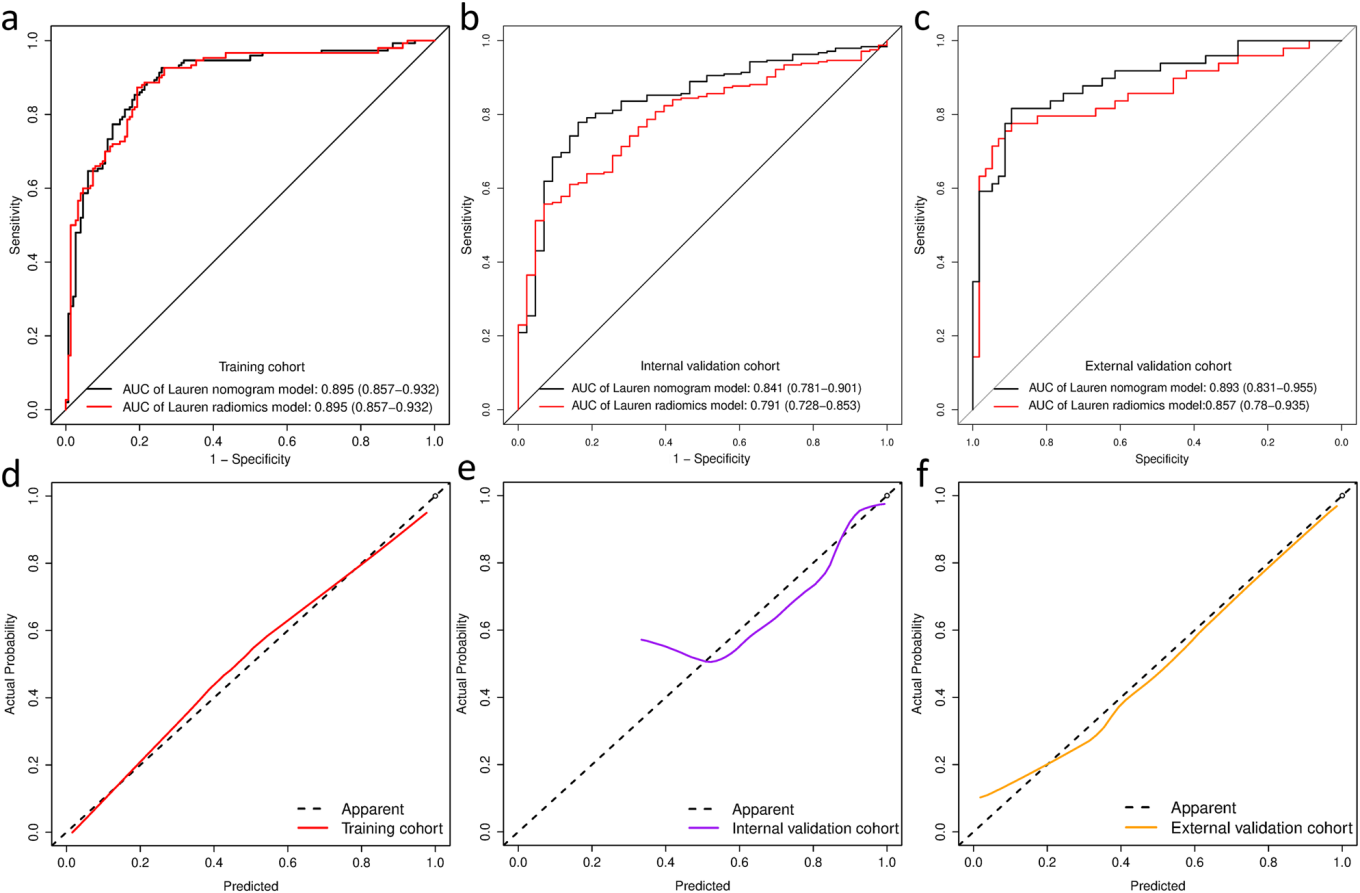
Evaluation of the Lauren radiomics nomograms. ROC curves comparing Lauren radiomics nomogram with Lauren radiomics SVM model in the training (**a**), internal validation (**b**) and external validation (**c**) cohorts. Calibration curves of the Lauren radiomics nomogram in the training (**d**), internal validation (**e**) and external validation (**f**) cohorts. *ROC* receiver operator characteristic, *SVM* support vector machine

The diagnostic performance comparison of the Lauren radiomics model and radiomics nomogram was shown in Fig. 3. No difference was observed in the training cohort (Fig. 3a), while the radiomics nomogram achieved a higher AUC than the radiomics model in the internal validation cohort (AUC, 0.841 [95%CI0.781–0.901] vs 0.791 [95%CI 0.728–0.853]) and external validation cohort (AUC, 0.893 [95%CI 0.831–0.955] vs 0.857 [95%CI 0.78–0.935]) (Fig. 3b and 3c). The Lauren radiomics nomogram model had higher specificity, sensitivity and accuracy than the SVM model (Additional file 1: Table S5**)**. The Delong test was applied on the ROC curves of the radiomics nomogram to assess possible overfitting and the result revealed that the differences were not statistically significant among the AUCs of the training cohort and the two validation cohorts, with P values of 0.138 and 0.969, respectively. The calibration curves demonstrated good agreement between prediction and observation in all three cohorts (Hosmer–Lemeshow test, *p* > 0.05) (Fig. 3d–f). The decision curve analysis (Additional file 1: Figure S6) indicated that the patients would benefit more from using the radiomics nomograms than using the SVM model or treat-all-patients scheme or the treat-none scheme if the threshold probability in the clinical decision was between 10 and 90%.

### Clinical characteristics of patients with diffuse-type GC

To further develop the SRCC radiomics model to distinguish SRCC and non-SRCC in diffuse-type GC patients, 394 diffuse-type GC patients from center 1 and 49 patients from center 2 were enrolled. The clinicopathologic characteristics of these patients were listed in Table 3. Clinical characteristics, including tumor location, differentiation status, Borrmann type, levels of CEA and CA199, and TNM stages were significantly different between non-SRCC and SRCC GC patients.

**Table 3:** Univariate and multivariate regression analysis of clinical characteristics in the training cohort of Lauren radiomics model and SRCC radiomics model

Lauren radiomics model Characteristics	Univariate analysis			Multivariate analysis		
	Odds ratio	95%CI	*p* value	Odds ratio	95%CI	*p* value
Age	0.977	(0.957, 0.997)	0.025	0.979	(0.958, 1.000)	0.049
Sex	1.425	(0.884, 2.299)	0.146	–	–	–
Tumor size	1.015	(1.001, 1.029)	0.033	–	–	–
Tumor location	1.417	(1.098, 1.827)	0.007	1.347	(1.035, 1.753)	0.027
CEA	2.359	(1.464, 3.802)	< 0.001	2.302	(1.417, 3.740)	0.001
CA199	2.224	(1.382, 3.578)	0.001	–	–	–

SRCC radiomics model Characteristics	Univariate analysis			Multivariate analysis		
	Odds ratio	95%CI	*p* value	Odds ratio	95%CI	*p* value
Age	0.984	(0.963, 1.006)	0.159	–	–	–
Sex	2.098	(1.267, 3.474)	0.004	2.044	(1.228, 3.404)	0.006
Tumor size	0.996	(0.986, 1.006)	0.387	–	–	–
Tumor location	1.472	(1.081, 2.004)	0.014	1.449	(1.058, 1.984)	0.021
CEA	0.931	(0.570, 1.520)	0.775	–	–	–
CA199	0.894	(0.548, 1.461)	0.655	–	–	–

*CEA* carcinoembryonic antigen, *CA19-9* carbohydrate antigen 19-9, *SRCC* signet ring cell carcinoma

### Feature selection and construction of the SRCC radiomics SVM model

**Fig. 4:**
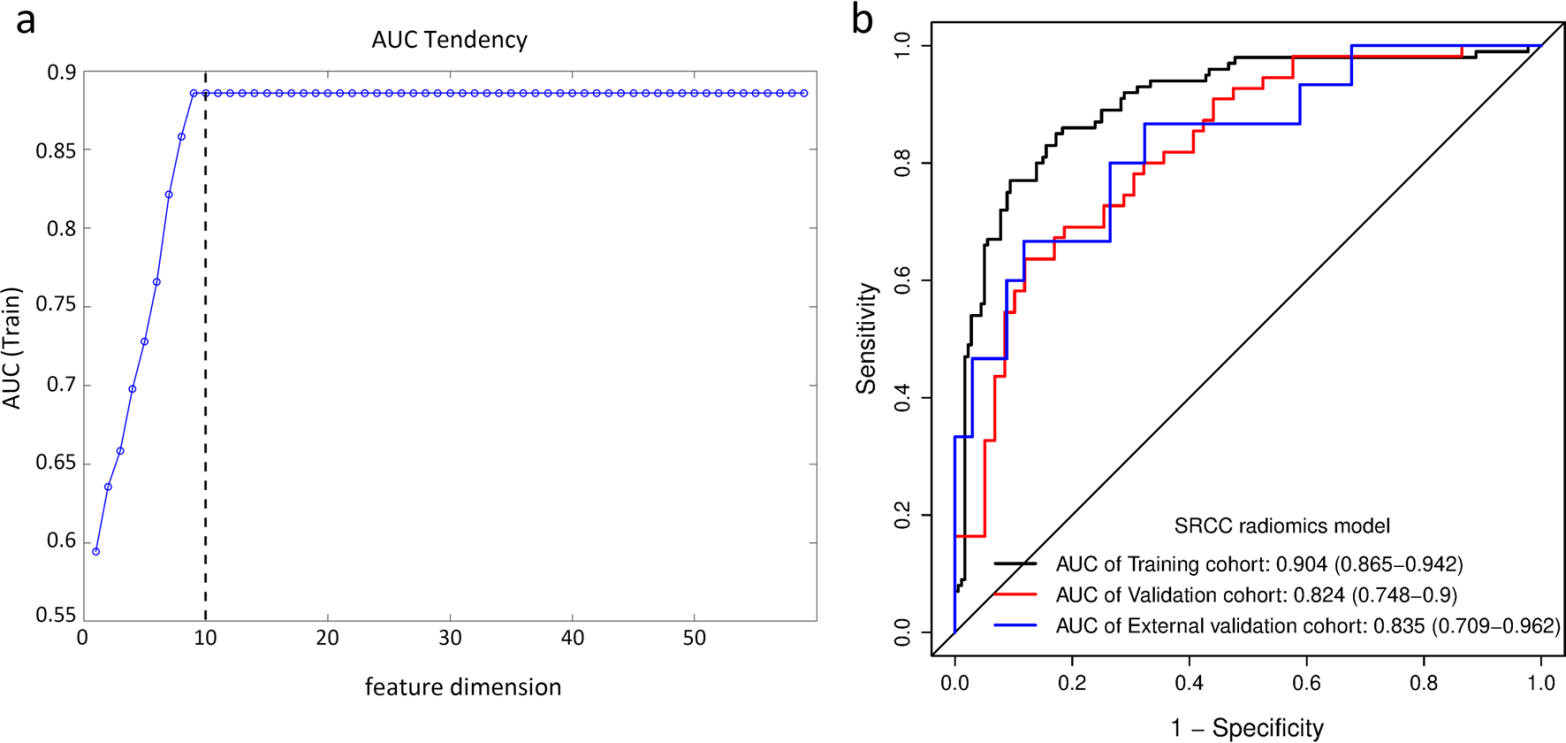
Construction of the SRCC radiomics SVM model. **a** The optimal feature subset of the SRCC radiomics SVM model included 10 features. **b** ROC curves of SRCC radiomics SVM model in the training, internal validation and external validation cohorts. *SRCC* signet ring cell carcinoma, *ROC* receiver operator characteristic, *SVM* support vector machine

Following the same feature selection and SVM model building procedure, we searched the feature subset with the best distinguishing characteristics for the SRCC radiomics model. Finally, the optimal feature subset with 10 features achieved excellent performance in distinguishing SRCC and non-SRCC patients (Fig. 4a and Additional file 1: Table S6), yielding AUC values of 0.904 (95%CI0.865–0.942), 0.824 (95%CI 0.748–0.9) and 0.835 (95%CI 0.709–0.962) in the training, internal and external validation cohort, respectively (Fig. 4b). Multivariate analysis revealed that Rad-score was the significant predictor of SRCC (OR, 6.193; 95%CI, (4.123, 9.303); *p* < 0.001) (Additional file 1: Table S4). Further, statistical difference was found in terms of 5-year DFS and OS between the high-Rad-score (SRCC) and low-Rad-score (non-SRCC) groups (Additional file 1: S9**)**.

### Construction and evaluation of the SRCC radiomics nomogram

**Fig. 5:**
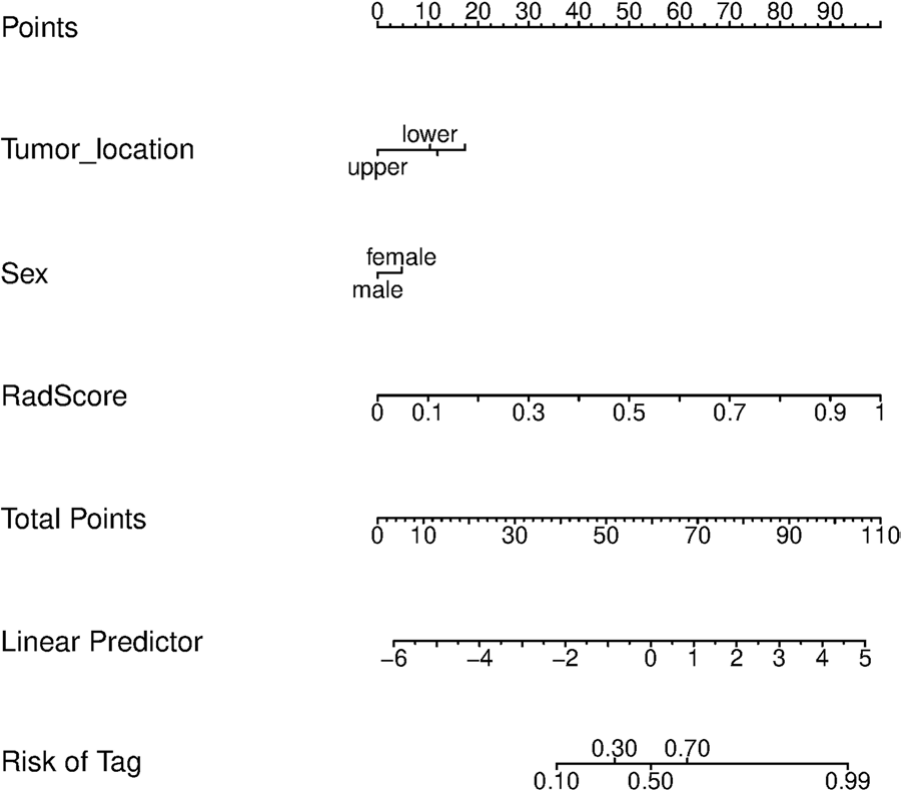
Development of the SRCC radiomics nomograms

Multivariate logistic regression analyses revealed that sex (OR, 2.044 [1.228, 3.404], *p* = 0.006) and tumor location (OR, 1.449 [1.058, 1.984], *p* = 0.021) were independent predictors between the SRCC and non-SRCC patients **(**Table 3). The Rad-score and clinical characteristics were incorporated to build the SRCC radiomics nomograms (Fig. 5).

**Fig. 6:**
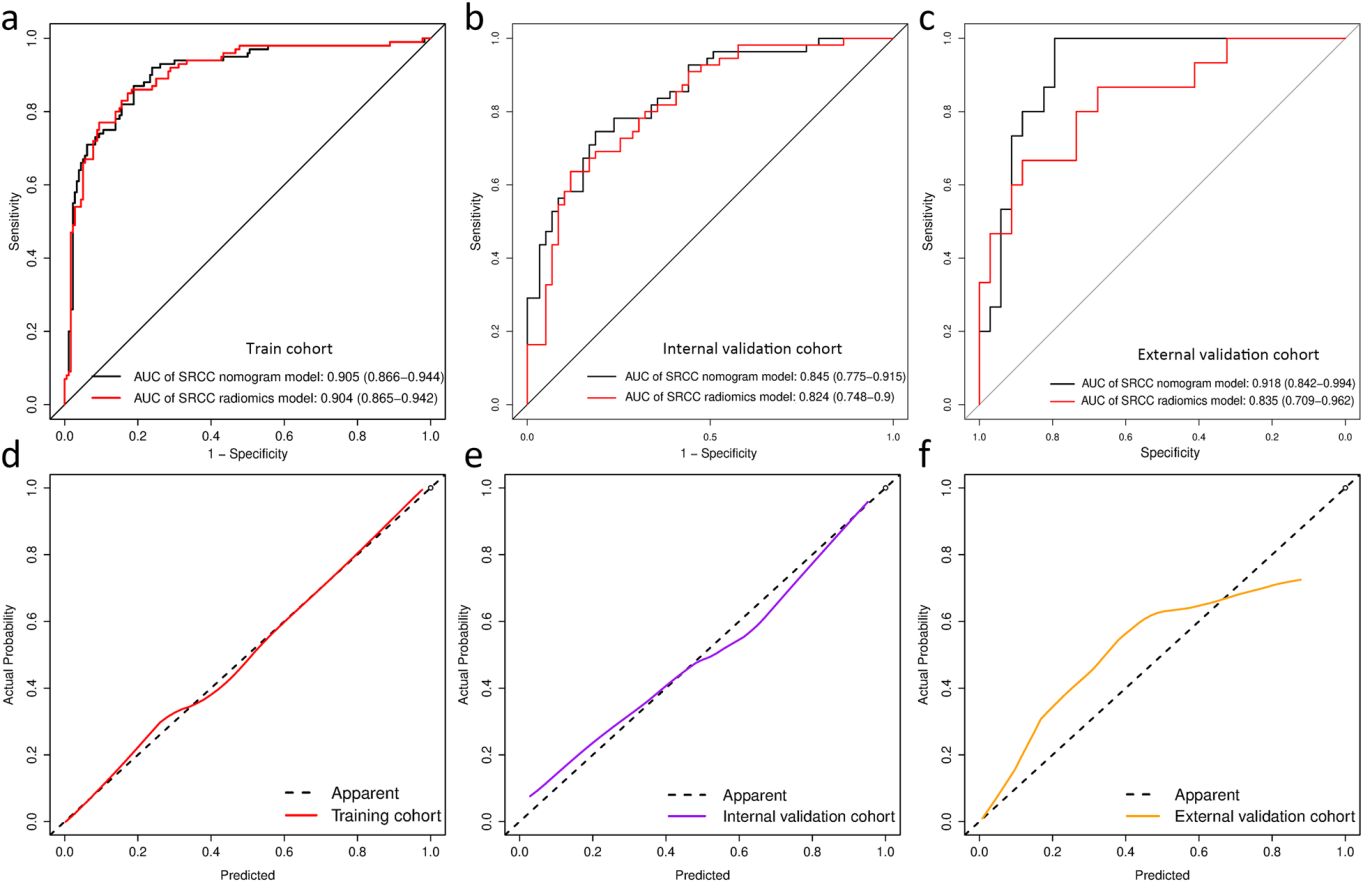
Evaluation of the SRCC radiomics nomograms. ROC curves comparing SRCC radiomics nomogram with SRCC radiomics SVM model in the training (**a**), internal validation (**b**) and external validation (**c**) cohorts. Calibration curves of the SRCC radiomics nomogram in the training (**d**), internal validation (**e**) and external validation (**f**) cohorts. *SRCC* signet ring cell carcinoma, *ROC* receiver operator characteristic, *SVM* support vector machine

The diagnostic performance comparison of the SRCC radiomics model and radiomics nomogram was shown in Fig. 6. No obvious differences were observed in the training cohort (Fig. 6a), while the radiomics nomogram achieved higher AUC than the radiomics model in the internal validation cohort (AUC, 0.845 [95%CI 0.775–0.915] vs 0.824 [95%CI 0.748–0.900]) (Fig. 6b) and external validation cohort (AUC, 0.918 [95%CI 0.842–0.994] vs 0.835 [95%CI 0.709–0.962]) (Fig. 6c). The SRCC radiomics nomogram model had higher specificity, sensitivity and accuracy than the SVM model (Additional file 1: Table S7**)**. The Delong test revealed that the differences were not statistically significant among the AUCs of the training cohort and the two validation cohorts, with P values of 0.138 and 0.969, indicating no overfitting was assessed. The calibration curves of the radiomics nomogram demonstrated good agreement between prediction and observation in all three cohorts (Hosmer–Lemeshow test, *p* > 0.05) (Fig. 6d–f). The decision curve analysis indicated that the patients would benefit more from using the radiomics nomograms than using the SVM model or treat-all-patients scheme or the treat-none scheme if the threshold probability in the clinical decision was between 10 and 90% (Additional file 1: S9).

## Discussion

In this retrospective multicenter study, we established a CT-based Lauren radiomics nomogram to identify the diffuse-type GC from all GC patients and further developed a SRCC radiomics nomogram to identify SRCC from diffuse-type GC. The nomograms provided a noninvasive and efficient preoperative diagnosis method to identify diffuse-type and SRCC GC.

Lauren classification is one of the most widely used histopathological classification systems for gastric adenocarcinoma [5, 26]. In addition to reflecting tumor biological behavior, it can also reflect the etiology, pathogenesis and epidemic characteristics of GC. Diffuse-type GC, which originates from the gastric mucosa and exhibits a diffuse growth pattern, is poorly differentiated and shows more chemotherapy resistance [27]. It is more prone to lymph node metastasis and distant metastasis than intestinal-type, resulting in a poor prognosis [28]. Studies have found that germline mutations in some genes (such as CDH1, BRCA2, STK11, ATM and PALB2) may be the cause of diffuse-type GC [14, 29, 30]. According to epidemiological data, there has been an increasing trend in the incidence of diffuse-type GC [3]. As a result, the early diagnosis and treatment of diffuse-type GC have attracted widespread attention worldwide. Gastroscopy and tissue biopsy are the most commonly used methods for the pathological diagnosis of GC. However, they are invasive operations, and the consistent rate of the Lauren classification was only 64.7% between biopsy and surgical samples [16]. The recent emergence of radiomics undoubtedly provides an excellent solution to this problem.

In this study, 693 GC patients from 2 centers were retrospectively analyzed, and 9691 radiomics features were extracted from their CT image. Radiomics feature subset with the best distinguishing characteristics was searched by SVM classifier to develop the Lauren radiomics model. SVM is a mature machine learning method with relatively stable performance and gradually replaces the previous lasso regression method. Multivariate analyses revealed that radiomics feature score could be the independent predictor of diffuse-type GC. Then, the Lauren radiomics nomogram integrating Rad-score and clinicopathological characteristics was developed, which was proved a promising AUC value and satisfactory calibration. Age, tumor size and CEA levels were found significantly associated with diffuse-type GC in this study, consistent with our previous literature review [16, 31].

SRCC, as a particular type of diffuse-type GC, is characterized by a higher incidence in females and a lower average age at diagnosis than non-SRCC [32]. Meanwhile, it has a higher rate of peritoneal carcinomatosis, lymph node invasion and chemotherapy resistance and a lower curative resection rate than non-SRCC tumors in advanced stages [2, 9, 14, 33]. Moreover, SRCCs often manifest as Borrmann IV type with a high false-negative rate during biopsy [17]. Considering the importance of early diagnosis, we further develop another radiomics SVM model (SRCC radiomics model) to identifying SRCC from diffuse-type GC. Multivariate analyses revealed that the model’s Rad-score could be the independent predictor of SRCC. Further, the SRCC nomogram integrating Rad-score and clinicopathological characteristics including sex and tumor location was developed. The results showed that the SRCC radiomics nomogram had higher AUC values and accuracy than the radiomics SVM model and the decision curve analysis demonstrated that the radiomics nomogram was clinically valuable.

In addition, nomograms in this study may also help future clinical decision making. Different pathological types of GC have different benefits from the same treatment, so it is necessary to choose appropriate treatment measures according to pathological types. For example, as for surgical management, diffuse-type GC usually need wider surgical margins to achieve an R0 resection, and a super-extended lymphadenectomy might be the best surgical approach [34]. A survival benefit with D3 lymphadenectomy, compared with D2 lymphadenectomy, can be obtained in diffuse-type and mixed-type GC [35]. In addition, diffuse-type GC may benefit from prevention and/or treatment of peritoneal metastases using hyperthermic intraperitoneal chemotherapy (HIPEC) [34, 36]. Therefore, if diffuse-type GC can be diagnosed and distinguished in an early stage, it will be of great help to the choice of treatment schemes and the prognosis evaluation.

There are some limitations to our study. First, as it was a retrospective study involving only two centers, further prospective research in more centers is needed to verify the radiomics nomograms. Second, SRCC is a special histological type with different clinical outcomes, depending on whether it is in an early or advanced stage [12, 18]. However, in this study, we did not perform analysis on this issue, only focused on the diagnosis of SRCC. Further radiomics research with subgroup analysis should be performed to reveal more biological characteristics of SRCC.

## Conclusion

In summary, we established two CT-based radiomics nomograms to identify the diffuse-type and SRCC GC, providing a noninvasive, efficient and preoperative diagnosis method. They may help guide preoperative clinical decision-making and benefit GC patients in the future.

## Supplementary Information

**Additional file 1.**

## Data Availability

All data generated or analyzed during this study are included in this published article.

## Abbreviations

GC: Gastric cancer
SRCC: Signet ring cell carcinoma
SVM: Support vector machine
ROI: Region of interest
ICCs: Inter- and intraclass correlation coefficients
RFS: Relief forward selection
ROC: Receiver operating characteristic
AUC: Area under the curve
DFS: Disease-free survival
OS: Overall survival
Rad-score: Radiomics feature score
CI: Confidence interval
OR: Odds ratio
HER2: Human epidermal growth factor 2
SEER: Surveillance Epidemiology and End Results
