# Supplementary material for: CT-based radiomics nomograms for preoperative prediction of diffuse-type and signet ring cell gastric cancer: a multicenter development and validation cohort
Source: J Transl Med. 2022 Jan 24;20:38. doi: 10.1186/s12967-022-03232-x (PMC8785479; doi:10.1186/s12967-022-03232-x)
Supplement: Supplementary file 1 — Additional file 1. [file 12967_2022_3232_MOESM1_ESM.docx]

**CT-based Radiomics Nomograms for Diagnosis and Prognosis Prediction of Intestinal-type, Diffuse-type and Signet ring cell Gastric Cancer: A Multicenter Development and Validation Cohort**

**Chen et al.**

**Supplementary Patients and Methods**

**Supplementary S1:** **The sample size consideration**

We retrospectively chose 300 consecutive patients treated in center 1 between December 2007 and March 2016 as training cohort in Lauren radiomics model. Considering that logistic regression performs best when case-to-noncase ratio is 1:1, we designed the training cohort to contain 150 diffuse-type GC patients and 150 intestinal-type GC patients who were randomly selected from the 394 diffuse-type GC patient pool. There were in total 4 predictors in Lauren radiomics nomogram, making an event-per-predictor ratio of 10. Therefore, we believed that there was no big concern on the overfitting issue of our model.

To further develop the SRCC radiomics model, we retrospectively chose 394 consecutive diffuse-type GC patients from center 1. To get better performance of the model, patients were randomly distributed into the training (280 of 394(71.1%)) and validation (114 of 394 (28.9%)) cohorts in a 7:3 ratio to make sure there were enough samples in the training cohort. In training cohort, there were 100 SRCC patients from the 155 SRCC patients pool. There were in total 3 predictors in SRCC radiomics nomogram, making an event-per-predictor ratio of 10. Therefore, we believed that there was no big concern on the overfitting issue of our model.

**Supplementary S2: CT Image Acquisition, Retrieving Procedure**

The patients from **Nanfang Hospital** underwent contrast-enhanced abdominal CT using multidetector-row CT (MDCT) systems (SIEMENS 64 slice spiral CT or GE Lightspeed 16, GE Healthcare). The acquisition parameters were as follows: tube voltage 120 kV; tube current 160-300 mAs; tube rotation time 0.5-0.8 s; detector collimation: 8×2.5 mm or 64×0.625 mm; field of view, 350×350 mm; matrix, 512×512. Contrast-enhanced CT scans were obtained at 25-30 s (arterial phase), 60 s (portal phase) and 180 s (delayed phase) following intravenous administration of 90-100 ml iodinated contrast material (Ultravist 370, Bayer Schering Pharma) at a rate of 3.0 or 3.5 ml/s with a pump injector (Ulrich CT Plus 150, Ulrich Medical). Contrast-enhanced CT images were reconstructed with a reconstruction thickness of 2-7.5 mm.

The cases in **Zhujiang Hospital** underwent contrast-enhanced abdominal CT using the MDCT systems (Philips Brilliance 64-MDCT scanner, Philips Medical Systems). The acquisition parameters are as follows: tube voltage 120 kV; tube current 200 - 300 mAs; tube rotation time 0.5s; field of view 350 × 350mm; matrix 512 × 512. Contrast-enhanced CT scans were obtained 25 - 30s (arterial phase), 60 s (portal phase) and 180s (delayed phase) following an injection of 80 - 100 mL of non-ionic iopamiro, a vascular contrast agent, at a rate of 5.0 ml/s. Contrast-enhanced CT was reconstructed with a reconstruction thickness of 5 mm.

**Supplementary S3: Delineation of tumor ROIs, reproducibility evaluation of intraobserver and interobserver**

Two clinical doctors (reader 1, W.X. and reader 2, T.C.) with more than 10 years of experience interpretating abdominal CT reviewed the CT image and delineated the tumor ROIs. Interobserver reliability and intraobserver reproducibility of feature extraction were analyzed by evaluating the differences between the features generated by two readers (interobserver), as well as the differences between the twice-generated features by reader 1 (intraobserver). Inter- and intraclass correlation coefficients (ICCs) were used to evaluate the agreement of features extraction. A good agreement was considered when the ICC was greater than 0.75. The readers were blinded to clinical information and pathologic results when they reviewed the CT images.

**Supplementary S4: Feature Analysis Methodology**

***Image preprocessing before texture feature extraction***

(1) Wavelet bandpass filtering: To reduce the noise in ROIs that interferes with texture information, this operation was carried out by applying different weights to bandpass and subbands (LHL, LHH, LLH, HLL, HHL and HLH) of the tumor region compared to low-frequency and high-frequency subbands (LLL and HHH) in the wavelet domain.

(2) Isotropic resampling: To maintain rotation invariance, in cases where the pixel and slice thickness of the ROI were fixed and the same in different directions, we resampled the ROI using cube interpolation to obtain the appropriate resolution.

(3) Quantization of gray level: We specified the compression or extension of the pixels to the gray level since all higher-order features involve a distance parameter. Moreover, quantization of the gray level was applied to normalize images with different attenuation values.

***Feature generation***

A series of texture features were generated from the images before/after preprocessing and are listed in Supplementary Table S2.

**Supplementary S5: Reduction of feature dimension**

According to the feature selection method in Chapter 11 of Machine Learning by Professor Zhou Zhihua of Nanjing University, the feature values were preprocessed with a filtering feature selection method.

(1) The training set samples, {(x_1_, Y_1_)..., (x_m_, y_m_)}, were read.

(2) The distance algorithm (min's distance was used here) was applied to calculate the nearest neighbor sample Xi, NH (NH is the abbreviation for near hit) in the same kind of samples (i.e., the same sample as Yi).

(3) Similarly, the nearest neighbor Xi, nm in the heterogeneous samples was obtained.

(4) After the completion of traversal, we obtained each sample and its two nearest neighbors and calculated the statistic $\delta^{j}$^j^

$$\delta^{i}=\sum_{i}^{m} -diff{(x_{i}^{j},x_{i,nh}^{j})}^{2}+diff{(x_{i}^{j},x_{i,nm}^{j})}^{2}$$

where the function diff (a, b) = | A-B |, $\delta^{j}$is the sum of the values of the$j^{th}$ component of the statistic $\delta$ for all samples.

(5) Given the threshold value $\tau$, all the component indexes of $\delta$ greater than $\tau$ were recorded, and the original features were retained according to this index.

**Supplementary S6: Cost SVM for classification(C-SVC)**

Support vector machine (SVM) is the statistical learning theory which solves the contradiction between the complexity and generalization of the model classifier through the structural risk minimization criterion and the kernel function method. In some real tasks, it is often difficult to find the appropriate kernel function to make the training cases linearly separable in the new eigenspace. C-SVC can tolerate error of classification, but cases that disobey the constraint should be as small as possible in condition of maximizing margin.

**Supplementary S7:** **Feature selection and construction of the radiomics models**

(1) The Relief algorithm was used to obtain each feature's weight, sort the feature weights from high to low, and obtain the sorted feature set represented by S.

(2) According to the order in S, starting with the first feature, the features were added to the classifiers one by one. The area under the curve (AUC) value was used as the index to evaluate the predictive performance of the classifiers.

(3) The features that improved the performance of the classifier were added to the feature subset, and the Relief algorithm was applied to the feature subset again (repeating steps 1 and 2) until the dimension of the feature subset did not decrease.

(4) Then, each feature in the feature subset was used as the promoter, and the feature that led to the highest performance of the classifier was selected as the real promoter. The second feature that combined with the promoter to yield the highest classification performance was set as the second feature, and so on.

**Supplementary S8: The Result of Reproducibility Evaluation of Radiomics Feature Extraction with Interobserver and Intraobserver.**

This study achieved satisfactory inter- and intra-observer reproducibility of the radiomics features extraction. There is no statistically significant difference in the features neither between of the repetition of reader 1 (P values ranged from 0.835 to 0.874) nor between reader 1’s first-extracted features and those of the reader 2 (P values ranged from 0.846 to 0.897). The inter-observer ICC calculated on the basis of reader 1’s first-extracted features and those of the reader 2 ranged from 0.8677 to 0.9224. The intra-observer ICC calculated based on reader 1’s twice feature extraction ranged from 0.9031 to 0.9344. Therefore, all outcomes were based on the features extracted by reader 1.

**Supplementary S9:** **The predictive performance of the model in terms of 5-year DFS and OS**

Accordingly, patients were classified into high Rad-score group (diffuse-type GC) and low Rad-score group (intestinal-type GC) in Lauren radiomics model. We assessed the 5-year DFS and 5-year OS of the two groups by using Kaplan-Meier survival analysis. The results showed that high Rad-score group had a worse long-term outcome in both the training and validation cohorts. The mean 5-year DFS and mean OS times were 47.105 (95% CI 43.255-50.955) and 49.851 (95% CI 46.321-53.380) months for low Rad-score group, which were longer than the times of 38.481 (95% CI 34.451-42.511) and 41.922 (95% CI 38.181-45.664) months for high Rad-score group, and the differences were statistically significant (p=0.007 and p=0.006) (**Supplementary Figure S4a and S4b**).

For SRCC radiomics model, high Rad-score group (SRC diffuse-type GC) showed worse long-term outcomes than low Rad-score group (non-SRC diffuse-type GC) in terms of DFS and OS. The mean 5-year DFS and OS times were 40.557 (95% CI 36.623- 44.491) and 44.975 (95% CI 41.426- 48.523) months for low Rad-score group, which were longer than the times of 34.030 (95% CI: 29.132- 38.928) and 37.666 (95% CI 33.010-42.321) months for high Rad-score group; the differences were statistically significant (p=0.021 and p=0.013) (**Supplementary Figure S4c and S4d**). We then performed the same analyses in the validation cohort, and similar results were observed (**Supplementary Figure S5**). Due to the missing data on long-term outcomes from the patients of Zhujiang Hospital Center, survival analysis was not performed on the external validation cohort.

**Supplementary Figures and Tables**

Preoperative standard CT available

n = 647

Patients with complete clinicopathologic data

n = 587

Preoperative standard CT available

n = 109

Patients with complete clinicopathologic data

n =106

The patient clinicopathologically diagnosed gastric cancer who underwent surgery with curative intent from December 2007 to March 2016 in center 1

n = 1946

The patient clinicopathologically diagnosed gastric cancer who underwent surgery with curative intent from January 2011 to December 2015 in center 2

n = 356

Training cohort

n =300

External validation cohort

n = 106

Internal validation cohort

n = 287

The patient with clinicopathologically diagnosed diffuse-type GC in center 1

n = 394

The patient with clinicopathologically diagnosed diffuse-type GC in center 2

n = 49

Training cohort

n =280

External validation cohort

n = 49

Internal validation cohort

n = 114

a

b

**Supplementary Figure S1:** **Flow Diagram for Eligible Patients**


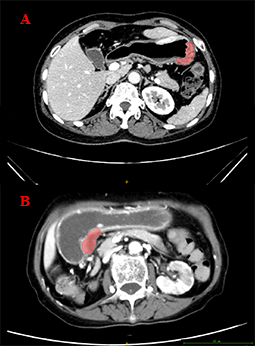


**Supplementary Figure S2.** **Manual segmentation of the ROI.** (A) CT image of a patient with SRC diffuse-type GC. (B) CT image of a patient with intestinal-type GC.

Abbreviations: ROI, region of interest; CT, computed tomography; SRC, signet ring cell; GC, gastric cancer.


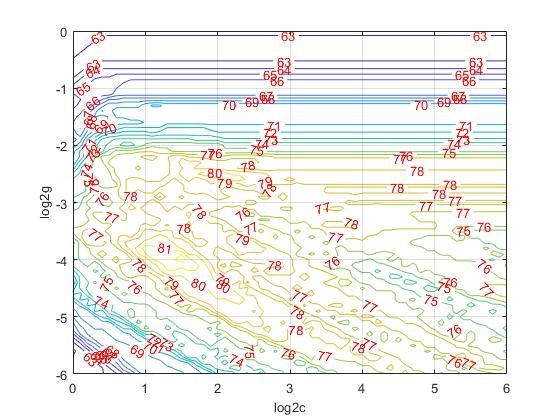


**Supplementary Figure S3.** **The tenfold cross-validation and grid search method to find the best combination of SVM model parameters c and g.**


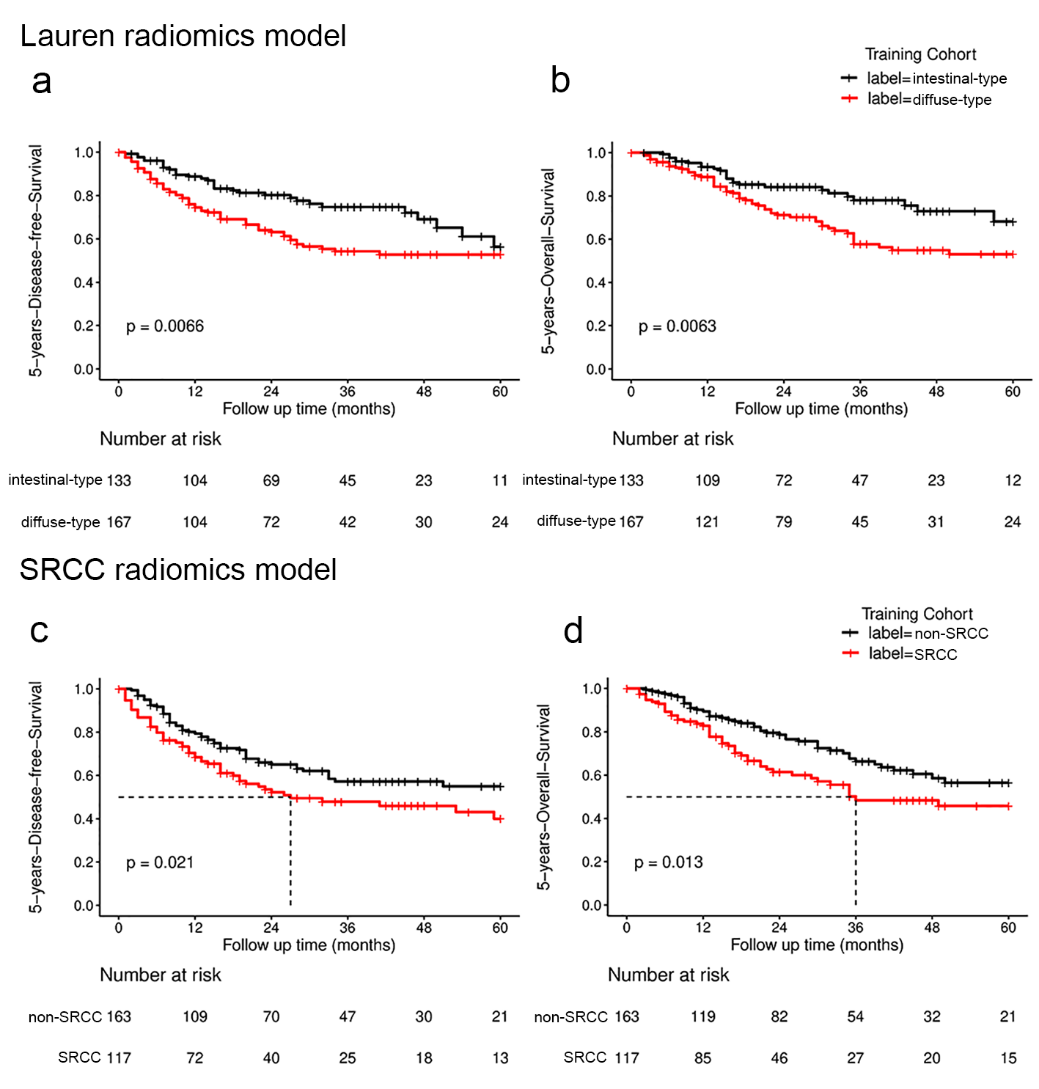


**Supplementary Figure S4. Kaplan-Meier survival in the training cohorts of Lauren and SRCC radiomics models.** (a) 5-years DFS of Lauren radiomics model. (b) 5-years OS of Lauren radiomics model. (c) 5-years DFS of SRCC radiomics model. (d) 5-years OS of SRCC radiomics model. *P*-values were calculated using the log-rank test.

Abbreviations: DFS, disease free survival; OS, overall survival.


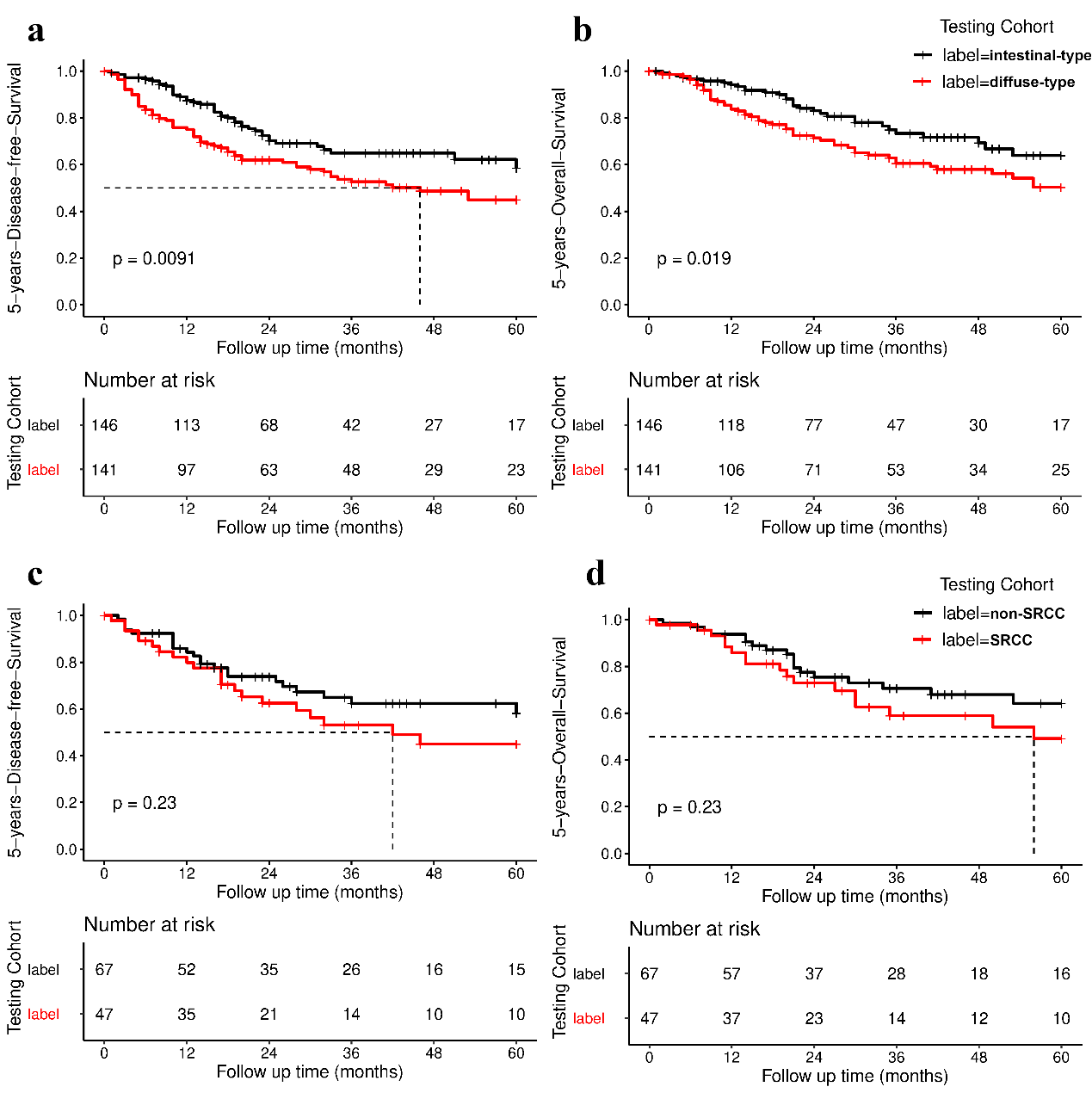


**Supplementary Figure S5. Kaplan-Meier survival in the validation cohorts of Lauren and SRCC radiomics models.** (a) 5-years DFS of Lauren radiomics model. (b) 5-years OS of Lauren radiomics model. (c) 5-years DFS of SRCC radiomics model. (d) 5-years OS of SRCC radiomics model. *P*-values were calculated using the log-rank test.

Abbreviations: disease free survival; OS, overall survival.


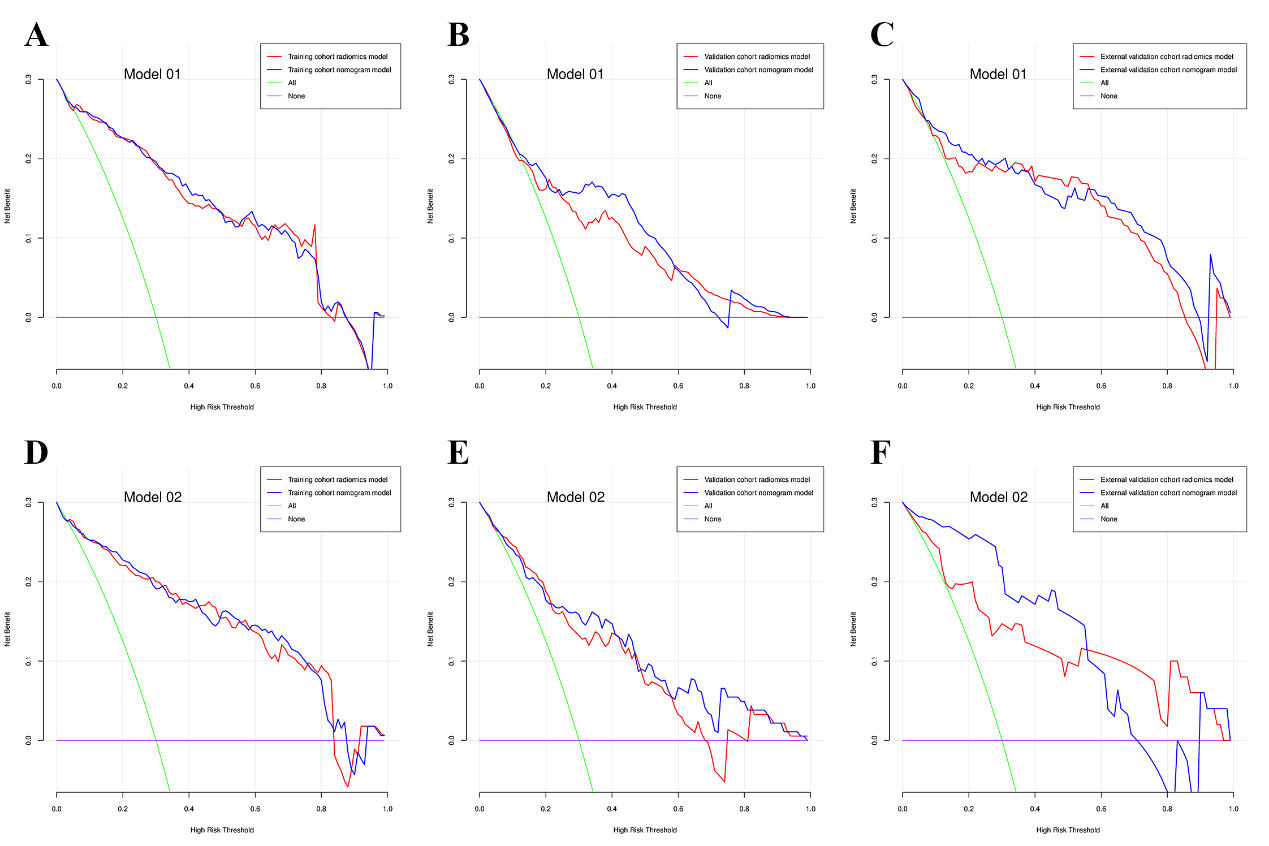


**Supplementary Figure S6.** Decision curve analysis for radiomics models and radiomics nomograms in the training, validation and external validation cohort of Lauren model (Model 01) and SRCC model (Model 02). The net benefits versus the threshold probability are plotted. The x-axis represents the predicted threshold probability and the y-axis represents the net benefit. It is obvious from the diagram that the radiomics models and nomograms can offer more net benefits than the default schemes across the majority of the range of threshold probabilities in both sets

| Parameters name | Values |
| --- | --- |
| Wavelet band-pass filtering | R=[1/2, 2/3, 1, 3/2, 2] |
| Isotropic voxel size | Scale={in-pR, 1, 2, 3, 4, 5} |
| Quantization algorithm | Quan algo={Equal, Lloyd} |
| Number of gray level | Ng=[8, 16, 32, 64] |

**Supplementary** **Table S1:** Different parameters are selected to enrich features in feature extraction

Note. According to permutation and combination theory, total number of features was generated by different features with different parameters.

| Texture | Reference(s) | Texture name |
| --- | --- | --- |
| Global | — | Variance  Skewness  Kurtosis |
| GLCM | Haralick et al 1973^[1]^ | Energy  Contrast  Entropy  Homogeneity  Correlation  Sum Average  Variance  Dissimilarity  Auto Correlation |
| GLRLM | Galloway 1975^[2]^ | Short Run Emphasis(SRE)  Long Run Emphasis(LRE)  Gray-Level Non-uniformity(GLN)  Run-Length Non-uniformity(RLN)  Run Percentage(RP) |
|  | Chu et al 1990^[3]^ | Low Gray-Level Run Emphasis(LGRE)  High Gray-Level Run Emphasis(HGRE) |
|  | Dasarathy and  holder 1991^[4]^ | Short Run Low Gray-Level Emphasis(SRLGE)  Short Run High Gray-Level Emphasis(SRHGE)  Long Run Low Gray-Level Emphasis(LRLGE)  Long Run High Gray-Level Emphasis(LRHGE) |
|  | Thibault et al 2009^[5]^ | Gray-Level Variance(GLV)  Run-Length Variance(RLV) |
| GLSZM | Calloway 1975^[2]^  Thibault et al 2009^[5]^ | Small Zone Emphasis(SZE)  Large Zone Emphasis(LZE)  Gray-Level Non-uniformity(GLN)  Zone-Size Non-uniformity(ZSN)  Zone Percentage(ZP) |
|  | Chu et al 1990^[3]^  Thibault et al 2009^[5]^ | Low Gray-Level Zone Emphasis(LGZE)  High Gray-Level Zone Emphasis(HGZE) |
|  | Dasarathy and  holder 1991^[4]^  Thibault et al 2009^[5]^ | Small Zone Low Gray-Level Emphasis(SZLGE)  Small Zone High Gray-Level Emphasis(SZHGE)  Large Zone Low Gray-Level Emphasis(LZLGE)  Large Zone Low Gray-Level Emphasis(LZHGE) |
|  | Thibault et al 2009^[5]^ | Gray-Level Variance(GLV)  Zone-Size Variance(SZV) |
| NGTDM | Amadasun and  King 1989^[6]^ | Coarseness  Contrast  Busyness  Complexity  Strength |

**Supplementary Table S2:** A series of texture features were generated from the images without/after preprocessing.

Abbreviations: GLCM, Gray-level co-occurrence matrix; GLRLM, Gray-level run-length matrix; GLSZM, Gray-level size zone matrix; NGTDM, Neighborhood gray-tone difference matrix.

| No | Feature | Wavelet bandpass | Isotropic voxel size | Quantisation algorithm | Quantised grey level |
| --- | --- | --- | --- | --- | --- |
| 1 | GLCM/Correlation | 0.67 | 2mm | Lloyd | 32 |
| 2 | GLCM/AutoCorrelation | 0.67 | 5mm | Equal | 16 |
| 3 | GLSZM/SZLGE | 1.5 | 3mm | Equal | 64 |
| 4 | GLSZM/SZHGE | 0.67 | 4mm | Lloyd | 64 |
| 5 | GLRLM/GLV | 1.5 | 3mm | Lloyd | 64 |
| 6 | GLCM/AutoCorrelation | 1.5 | 3mm | Equal | 64 |
| 7 | GLSZM/SZHGE | 1 | 4mm | Lloyd | 64 |
| 8 | GLSZM/HGZE | 0.67 | 5mm | Equal | 16 |
| 9 | GLCM/AutoCorrelation | 0.5 | 5mm | Equal | 16 |
| 10 | GLRLM/RLV | 1 | 4mm | Lloyd | 16 |
| 11 | NGTDM/Complexity | 1 | 5mm | Equal | 16 |
| 12 | GLRLM/SRHGE | 1.5 | 5mm | Equal | 16 |
| 13 | GLSZM/ZP | 2 | 3mm | Equal | 64 |

**Supplementary Table S3:** Optimal feature subset of Lauren radiomics model.

Abbreviations: GLCM, Gray-level co-occurrence matrix; GLSZM, Gray-level size zone matrix; SZLGE, Small Zone Low Gray-Level Emphasis; SZHGE, Small Zone High Gray-Level Emphasis; GLRLM, Gray-level run-length matrix; GLV, grey-level variance; HGZE, High Gray-Level Zone Emphasis; RLV, Run-Length Variance; SRHGE, Short Run High Gray-Level Emphasis; NGTDM, Neighborhood gray-tone difference matrix; ZP, Zone Percentage.

| Lauren radiomics model Characteristics | Univariate analysis | | |  | Multivariate analysis | | |
| --- | --- | --- | --- | --- | --- | --- | --- |
|  | Odds ratio | 95% CI | *p* value |  | Odds ratio | 95% CI | *p* value |
| Age | 0.977 | (0.957, 0.997) | 0.025 |  | - | - | - |
| Sex | 1.425 | (0.884, 2.299) | 0.146 |  | - | - | - |
| Tumor size | 1.015 | (1.001, 1.029) | 0.033 |  | - | - | - |
| Tumor location | 1.417 | (1.098, 1.827) | 0.007 |  | - | - | - |
| CEA | 2.359 | (1.464, 3.802) | <0.001 |  | - | - | - |
| CA199 | 2.224 | (1.382, 3.578) | 0.001 |  | - | - | - |
| Rad-score | 4.164 | (3.121,5.557) | <0.001 |  | 4.164 | (3.121,5.557) | <0.001 |
| SRCC radiomics model Characteristics | Univariate analysis | | |  | Multivariate analysis | | |
|  | Odds ratio | 95% CI | *p* value |  | Odds ratio | 95% CI | *p* value |
| Age | 0.984 | (0.963, 1.006) | 0.159 |  | - | - | - |
| Sex | 2.098 | (1.267, 3.474) | 0.004 |  | - | - | - |
| Tumor size | 0.996 | (0.986, 1.006) | 0.387 |  | - | - | - |
| Tumor location | 1.472 | (1.081, 2.004) | 0.014 |  | - | - | - |
| CEA | 0.931 | (0.570, 1.520) | 0.775 |  | - | - | - |
| CA199 | 0.894 | (0.548, 1.461) | 0.655 |  | - | - | - |
| Rad-score | 6.193 | (4.123,9.303) | <0.001 |  | 6.193 | (4.123,9.303) | <0.001 |

**Supplementary Table S4:** Univariate and multivariate regression analysis of Rad-score and clinical characteristics in training cohort of Lauren radiomics model and SRCC radiomics model.

Abbreviations: CEA, carcinoembryonic antigen; CA19-9, carbohydrate antigen 19-9.

| Index of Lauren models | Training cohort | | Internal validation cohort | | External validation cohort | |
| --- | --- | --- | --- | --- | --- | --- |
|  | SVM model | Nomogram model | SVM model | Nomogram model | SVM model | Nomogram model |
| Speciﬁcity | 0.807 | 0.820 | 0.451 | 0.860 | 0.895 | 0.895 |
| Sensitivity | 0.833 | 0.820 | 0.953 | 0.971 | 0.776 | 0.816 |
| Accuracy | 72.11% | 82.00% | 71.43% | 84.67% | 82.01% | 85.85% |

**Supplementary Table S5:** The specificity, sensitivity and accuracy results of Lauren radiomics SVM model and nomogram model.

| No | Feature | Wavelet bandpass | Isotropic voxel size | Quantisation algorithm | Quantised grey level |
| --- | --- | --- | --- | --- | --- |
| 1 | GLSZM/GLN | 0.67 | 'pixelW' | Equal | 8 |
| 2 | GLSZM/HGZE | 1.50 | 1mm | Lloyd | 8 |
| 3 | GLSZM/SZLGE | 0.50 | 4mm | Lloyd | 64 |
| 4 | GLSZM/GLN | 1.00 | 1mm | Lloyd | 8 |
| 5 | GLSZM/GLN | 0.50 | 1mm | Lloyd | 16 |
| 6 | GLRLM/GLV | 0.50 | 5mm | Lloyd | 16 |
| 7 | GLSZM/HGZE | 0.50 | 2mm | Lloyd | 8 |
| 8 | GLRLM/LGRE | 0.50 | 3mm | Lloyd | 64 |
| 9 | GLSZM/LZLGE | 0.50 | 4mm | Equal | 32 |
| 10 | GLSZM/HGZE | 1.00 | 1mm | Lloyd | 8 |

**Supplementary Table S6:** Optimal feature subset of SRCC radiomics model.

Abbreviations: GLSZM, Gray-level size zone matrix; GLN, Gray-Level Non-uniformity; HGZE, High Gray-Level Zone Emphasis; SZLGE, Small Zone Low Gray-Level Emphasis; GLV, grey-level variance; HGZE, High Gray-Level Zone Emphasis; LGRE, Low Gray-Level Run Emphasis; LZLGE, Large Zone Low Gray-Level Emphasis.

| Index of SRCC models | Training cohort | | Internal validation cohort | | External validation cohort | |
| --- | --- | --- | --- | --- | --- | --- |
|  | SVM model | Nomogram model | SVM model | Nomogram model | SVM model | Nomogram model |
| Speciﬁcity | 0.883 | 0.878 | 0.712 | 0.763 | 0.912 | 0.912 |
| Sensitivity | 0.770 | 0.750 | 0.727 | 0.782 | 0.467 | 0.667 |
| Accuracy | 87.78% | 83.21% | 73.91% | 77.19% | 81.63% | 83.67% |

**Supplementary Table S7:** The specificity, sensitivity and accuracy results of SRCC radiomics SVM model and nomogram model.

**References**

[1] Haralick R M, Shanmugam K, Dinstein I. Textural Features for Image Classification. Studies in Media and Communication, 1973; SMC-3(6):610-621.

[2] Galloway M. Texture analysis using gray level run lengths. Computer Graphics & Image Processing, 1975; 4(2):172-179.

[3] Chu A , Sehgal C M , Greenleaf J F . Use of gray value distribution of run lengths for texture analysis. Pattern Recognition Letters, 1990; 11(6):415-419.

[4] Dasarathy B V, Holder E B. Image characterizations based on joint gray level-run length distributions. Pattern Recognition Letters, 1991; 12(8):497-502.

[5] Thibault G, Fertil B, Navarro C, et al. Shape and texture indexes and gray level size zone matrix application to cell nuclei classification. Int J Pattern Recogn. 2013; 27:155–167.

[6] Amadasun M, King R. Textural features corresponding to textural properties. IEEE Trans Syst, Man, Cybern. 1989; 19:1264–1274.
